# Electron Fluxes in Biocathode Bioelectrochemical Systems Performing Dechlorination of Chlorinated Aliphatic Hydrocarbons

**DOI:** 10.3389/fmicb.2018.02306

**Published:** 2018-09-28

**Authors:** Fan Chen, Zhiling Li, Jiaqi Yang, Bin Liang, Cong Huang, Weiwei Cai, Jun Nan, Aijie Wang

**Affiliations:** ^1^State Key Laboratory of Urban Water Resources and Environment, School of Environment, Harbin Institute of Technology, Harbin, China; ^2^Key Laboratory of Environmental Biotechnology, Research Center for Eco-Environmental Sciences, Chinese Academy of Sciences, Beijing, China

**Keywords:** bioelectrochemical systems, biocathode, chlorinated aliphatic hydrocarbons, dechlorination characteristics, electron fluxes

## Abstract

Bioelectrochemical systems (BESs) are regarded as a promising approach for the enhanced dechlorination of chlorinated aliphatic hydrocarbons (CAHs). However, the electron distribution and transfer considering dechlorination, methanogenesis, and other bioprocesses in these systems are little understood. This study investigated the electron fluxes in biocathode BES performing dechlorination of three typical CAHs, 1,1,2,2-tetrachloroethene (PCE), 1,1,2-trichloroethene (TCE) and 1,2-dichloroethane (1,2-DCA). Anaerobic sludge was inoculated to cathode and biocathode was acclimated by the direct acclimation and selection. The constructed biocathode at −0.26 V had significantly higher dechlorination efficiency (*E*_24h_ > 99.0%) than the opened circuit (*E*_24h_ of 17.2–27.5%) and abiotic cathode (*E*_24h_ of 5.5–10.8%), respectively. Cyclic voltammetry analysis demonstrated the enhanced cathodic current and the positive shift of onset potential in the cathodic biofilm. Under autotrophic conditions with electrons from the cathode as sole energy source (columbic efficiencies of 80.4–90.0%) and bicarbonate as sole carbon source, CAHs dechlorination efficiencies were still maintained at 85.0 ± 2.0%, 91.4 ± 1.8%, and 84.9 ± 3.1% for PCE, TCE, and 1,2-DCA, respectively. *Cis*-1,2-dichloroethene was the final product for PCE and TCE, while 1,2-DCA went through a different dechlorination pathway with the non-toxic ethene as the final metabolite. Methane was the main by-product of the heterotrophic biocathode, and methane production could be enhanced to some extent by electrochemical stimulation. The various electron fluxes originating from the cathode and oxidation of organic substrates might be responsible for the enhanced CAHs dechlorination, while methane generation and bacterial growth would probably reduce the fraction of electrons provided for CAH dechlorination. The study deals with the dechlorination and competitive bioprocesses in CAH-dechlorinating biocathodes with a focus on electron fluxes.

## Introduction

Groundwater contamination by chlorinated aliphatic hydrocarbons (CAHs), such as 1,1,2,2-tetrachloroethene (PCE), 1,1,2-trichloroethene (TCE) and 1,2-dichloroethane (1,2-DCA), have posed serious environmental and health concerns (Huang et al., 2014). The bioelectrochemical systems (BESs) with biocatalyzed reduction at the cathode have been regarded as a promising technology for the *in situ* remediations of the CAHs-contaminated environments (Lohner et al., 2011; Palma et al., 2018).

During the anaerobic bioremediation of contaminated sites, electron donors (organic substrates, H_2_, or electrodes) are added to stimulate the growth and metabolism of microbial populations capable of reductively dechlorinating CAHs (Rosenbaum et al., 2011; Hug et al., 2013). Organohalide respiration is the utilization of halogenated compounds as terminal electron acceptors in anaerobic respiration (Hug et al., 2013; Schubert et al., 2018). However, some non-organohalide respiration microorganisms, such as methanogens and denitrifying bacteria will also participate in electron competition, reducing the efficiency of electron utilization for organohalide respiration (Aulenta et al., 2011; Hug et al., 2013; Chen et al., 2019).

Several biocathode systems for CAHs dechlorination have been established with either dehalorespiring isolates, highly enriched consortia or anaerobic sludge (Aulenta et al., 2010; Strycharz et al., 2010; Wang et al., 2015a; Chen et al., 2018). In principle, biocathode systems aid the dechlorination process in supplying the sustained electrons and redox environment (Liang et al., 2013; Sun et al., 2015). For CAH-dechlorinating biocathodes, the ability and efficiency of dechlorinating bacteria to acquire electrons from the cathode and oxidation of organic substrate significantly influence the CAH-dechlorinating performance. The distribution of electrons in biocathode BES is crucial for CAH dechlorination (Freguia et al., 2007; Virdis et al., 2009). Several researches have recognized the role of electrochemical stimulation in regulating electron fluxes, the activity and the synergistic interactions of the microorganisms growing in the anode compartment of microbial fuel cells (Virdis et al., 2009; Wang et al., 2015b). However, to date, no report focused on the electron fluxes from the electrode or organic carbons to CAH dechlorination or methane generation in CAHs-dechlorinating biocathodes.

In this study, biocathode systems for dechlorination of three types of CAHs (PCE, TCE, and 1,2-DCA) were established by acclimating with anaerobic sludge. The dechlorination metabolites, pathways, competitive processes and quantification of electron transfer were thoroughly investigated during dechlorination of three typical CAHs. The objective of this study was to reveal the electron fluxes in biocathode BES performing the dechlorination of CAHs through comprehensive dechlorination pathways analysis and electron balance calculations.

## Materials and Methods

### System Construction

The BES reactors were constructed by assembling two equal-size cylindrical borosilicate glass chambers (4 cm in diameter and 8 cm in length with an effective volume of 80 mL) and separated by cation exchange membranes (CEM, Ultrex CMI-7000, Membranes International, Ringwood, NJ, United States) as described previously (Chen et al., 2018). The two chambers were fixed together by an aluminum clamp and rubber seal, which also ensured airtightness. Pretreated graphite fiber brushes (both 3 cm in diameter and length, TohoTenax Co., Ltd., Tokyo, Japan) served as both the anodic and cathodic electrodes. Titanium wires (1 mm in diameter, Baoji LiXing Titanium Group Co., Ltd., China) were used to make the connection between the electrodes and the external circuits. A saturated calomel electrode (SCE) with the potential of 0.247 V vs. standard hydrogen electrode (SHE; model-217, Shanghai Precise. Sci. Instru. Co., Ltd., China) was placed in the circuit. A potentiostat (model-660D, CH Instruments Inc., Austin, TX, United States) was applied for potential control and data acquisition. All potentials reported were with respect to SHE.

### System Acclimation and Operation

Cathode chamber was inoculated with a mixture of 10 mL of anaerobic sludge taken from anaerobic sludge thickener (Taiping wastewater treatment plant, Harbin, China). Whether the sludge was contaminated with any CAH was unknown. The initial concentrations of PCE (0.3 mM), TCE (1.0 mM), and 1,2-DCA (2.2 mM) were obtained based on their respective solubility in water and the concentrations applied in previous studies (Wang et al., 2015a). The catholyte solution consisting of one of the three CAHs, sodium acetate (5 mM), phosphate buffered saline (PBS, 5 mM, pH = 7), vitamin solution and trace element solution was also added at a volume of 50 mL following Li et al. (2016). The catholyte was anaerobically prepared by aerating N_2_ and filtration via a 0.22 μm filter during all the experiments. The anode chamber was filled up with 5 mM phosphate buffer solution (PBS, pH = 7).

A cathode potential of −0.26 V was applied in this study, representing a condition that dechlorination was almost driven by direct electrons transfer from electrode without abiotic H_2_-generation (Strycharz et al., 2008). Systems were initially incubated for 10 days by stimulating sludge with a weak potential of −0.26 V. Subsequently, the catholyte was discharged and the cathode chamber was replenished with fresh medium mixture. Then, the repeated inoculation of sludge was performed to continuously develop the cathodic biofilm. All applied solutions were sterile and anaerobic, and solution replacement was performed in an anaerobic operation box (YQX-II, Shanghai Yuejin Medical Instru. Co., Ltd., China). After five iterations, no sludge was added to the cathode chamber and the replacement of catholyte was conducted every 4–6 days after CAHs dechlorination activity was observed. This procedure was repeated for approximately 60 d to achieve the development of biofilm on the cathode. After that, the experiments were performed for seven running cycles of 32 h for test analysis.

CAHs dechlorination was tested under four different modes: (i) NaAc-fed biocathode system where the acetate and electrode served as internal and external electrons donors, respectively; (ii) NaHCO_3_-fed biocathode where the acetate was replaced with NaHCO_3_ to ensure that the electrons were solely collected from the electrode. Activity for NaHCO_3_-fed biocathode was determined after three times medium exchange to completely remove residual acetate from the system; (iii) opened circuit biocathode system where no negative potential was applied and the anaerobic sludge was inoculated in parallel; and (iv) abiotic cathode system where the negative potential was applied in parallel and no sludge was added. In the pre-experiment, the removal of the three CAHs by the heterotrophic cathode reached more than 99% at 24 h. In addition, the CAHs dechlorination efficiencies at 24 h also showed a significant difference under the different modes. Therefore, the samples at 24 h were selected for detailed test analysis. All prepared systems were operated in triplicate at an ambient temperature of 23 ± 3°C. To characterize the bioelectrochemical properties of the established systems, cyclic voltammetry (CV) analysis was performed using an electrochemical workstation (model-660D, CH Instruments Inc., Austin, TX, United States). Cyclic voltammograms were recorded with a low scanning rate of 1 mV/s at 25°C.

### Chemicals and Analytical Methods

1,1,2,2-tetrachloroethene (≥99.5%), 1,1,2-trichloroethene (99.5%), *trans*-1,2-dichloroethene (*trans*-1,2-DCE; ≥98%), 1,1-dichloroethene (1,1-DCE; ≥99%), *cis*-1,2-dichloroethene (*cis*-1,2-DCE; ≥97%), 1,2-dichloroethane (1,2-DCA; ≥99%) and vinyl chloride (VC; 100 μg/mL in methanol) were purchased from Sigma-Aldrich (St. Louis, MO, United States). All other chemicals used to prepare analytical standards or feed solutions were analytical reagent grade.

Two milliliters of liquid samples obtained from the cathode chambers were immediately transferred to sealed 10 mL bottles filled with the high purity N_2_ gas (≥99.99%). The bottles were placed in a 25°C shaker for 30 min to reach the equilibrium. Concentrations of volatile organic compounds including PCE, TCE, *trans*-1,2-DCE, 1,1-DCE, *cis*-1,2-DCE, 1,2-DCA, and VC in the headspace (8 mL) were determined using a gas chromatograph (Agilent 7890A, Palo Alto, CA, United States) equipped with a 63Ni electron capture detector and DB-1301 column (30 m × 250 μm × 0.25 μm, Agilent). Ethene and methane were determined using a gas chromatograph (Agilent 7890A, Palo Alto, CA, United States) equipped with flame ionization detector (FID) and HP-5 column (30 m × 250 μm × 0.25 μm, Agilent). Headspace concentrations were converted to aqueous-phase using tabulated Henry’s law constants (Chen et al., 2018).

### Calculations

The dechlorination efficiency of CAHs (*E*, %) was calculated using equation (1). The recovery rate of CAH dechlorination products (*R_p-t_*, %) was calculated using equation (2).

(1) $$E=\frac{C_{0}-C}{C_{0}}\times 100\%$$

(2) $$R_{p-t}=\frac{C_{p}}{C_{0}-C}\times 100\%$$

where *C* represents CAH concentration (mM) at time *t* (h), *C*_0_ represents the initial CAH concentration (mM), and *C*_p_ represents the concentration of dechlorination products (mM) of CAHs at time *t* (h).

The electron balances were calculated according to the sum of donated electrons and accepted electrons (mmol e^−^/L). The number of electrons donated from acetate was calculated by multiplying the consumed acetate concentration (mM) by eight, the number of mol of electrons generated during complete oxidation of 1 mol acetate to CO_2_ (CH_3_COOH→2CO_2_, −8e). Electrons donated from the electrode ([electrode]; mmol e^−^/L) were measured as moles of electrons flowing through the unit reaction solution, calculated using equation (3).

(3) $$[\mathrm{electrode}]=\frac{Q}{V\times F}\times 10^{3}$$

where *Q* denotes the electric quantity (C) transferred at the electrodes, calculated by integrating the current (A) over the period of electrode polarization (s); V is the empty volume of the cathode chamber (L); F is the Faraday’s constant (96485 C/mol electrons).

Electrons accepted by CAHs dechlorination were measured by summing up the products of the measured CAH dechlorination products (mM) and the number of moles of electrons for the formation of 1 mol of dechlorination products. Electrons accepted for methane generation were calculated by multiplying the effluent methane concentration (mM) by eight, the number of moles of electrons required for the formation of 1 mol of methane from CO_2_ reduction (CO_2_→CH_4_,+8e). Electron recovery (%) was calculated as the ratio of electrons accepted for CAHs dechlorination and methane generation and electrons donated from the electrode and consumed acetate.

Coulombic efficiency for PCE dechlorination (εPCE, %) was calculated as the ratio of the theoretical electric quantity used for the formation of dechlorination products to the electric quantity flowing across the biocathode according to equation (4).

(4) $$\mathrm{\varepsilon PCE}=\frac{(2\times [\mathrm{TCE}]+4\times [\mathrm{DCE}]+8\times [\mathrm{ethene}])\times V\times F}{Q}\times 100\%$$

where 2, 4, or 8 are the number of mole electrons required for the formation of 1 mol of TCE, DCE, or ethene from PCE, respectively; and [TCE], [DCE], and [ethene] are the concentrations (mol/L) of TCE dechlorination products in the cathode effluent. Similarly, coulombic efficiencies for TCE and 1,2-DCA dechlorination were calculated using equations (5) and (6).

(5) $$\mathrm{\varepsilon TCE}=\frac{(2\times [\mathrm{DCE}]+6\times [\mathrm{ethene}])\times V\times F}{Q}\times 100\%$$

(6) $$\mathrm{\varepsilon DCA}=\frac{(2\times [\mathrm{ethene}])\times V\times F}{Q}\times 100\%$$

## Results and Discussion

### CAHs Dechlorination Performances

**FIGURE 1:**
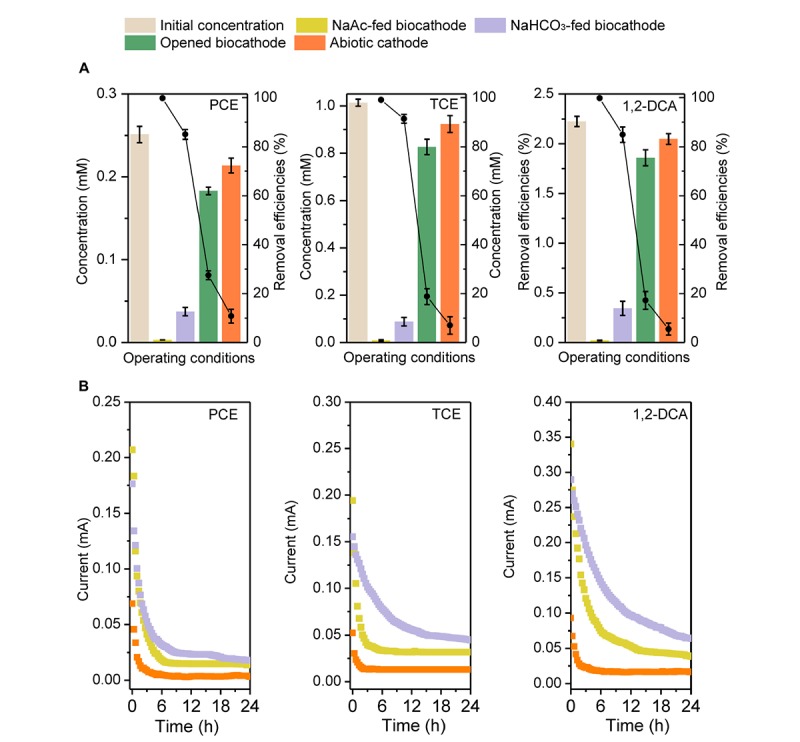
The concentration, removal efficiency **(A)**, and the dynamic current changes **(B)** of PCE, TCE, and 1,2-DCA under different operational conditions in biocathode system.

For the three CAHs, the biocathode had 2.1–4.8 and 6.9–17.2 times higher dechlorination efficiency (*E*_24h_ > 99.0%) than the opened circuit (*E*_24h_ of 17.2–27.5%) and abiotic cathode (*E*_24h_ of 5.5–10.8%), respectively, indicating the enhanced CAHs dechlorination performances with a biocatalyzed cathode (**Figure 1**). When biocathode was switched from heterotrophic to autotrophic (NaHCO_3_-fed) cultivation without external electron donor, *E*_24h_ could still be maintained at 85.0 ± 2.0%, 91.4 ± 1.8% and 84.9 ± 3.1% for PCE, TCE, and 1,2-DCA, respectively. This verified that the cathodic biofilm could utilize electrode as the sole electron donor for CAHs dechlorination and use these electrons to build up a transmembrane electrochemical gradient (Rabaey and Rozendal, 2010; Liang et al., 2014; Sun et al., 2016). The NaAc- and NaHCO_3_-fed biocathode had the same cathodic potential of −0.26 V and the initial reaction currents of both were similar (**Figure 1**). However, in 24 h of testing, the reaction current of NaAc-fed biocathode decreased to less than 0.03 mA, while NaHCO_3_-fed biocathode had 22.1–66.5% of higher current values than NaAc-fed biocathode. Current variation indicated the possible stronger involvement of cathodic electron transfer process in the CAHs dechlorination under autotrophic biocathode. On the other hand, *E*_24h_ in the NaHCO_3_-fed mode was 8.4–17.6% times lower than that of the acetate-fed biocathode (**Figure 1**). Previous reports have verified that acetate can serve as an electron donor for dechlorinators or methanogens or as a carbon source for cell synthesis and growth (He et al., 2002). Strains TT4B, MS-1, BB1, and BRS1 have been reported to couple acetate oxidation to PCE dehalogenation (Krumholz et al., 1996; Sharma and McCarty, 1996; Sung et al., 2003). Strain SF3 can grow by coupling the reductive dechlorination of 2-chlorophenol to the oxidation of acetate (Sun et al., 2000). As such, the contributions of sodium acetate to the dechlorination process were further discussed in section 3.4.

### Catalytic Activities of the Cathodic Biofilm

**FIGURE 2:**
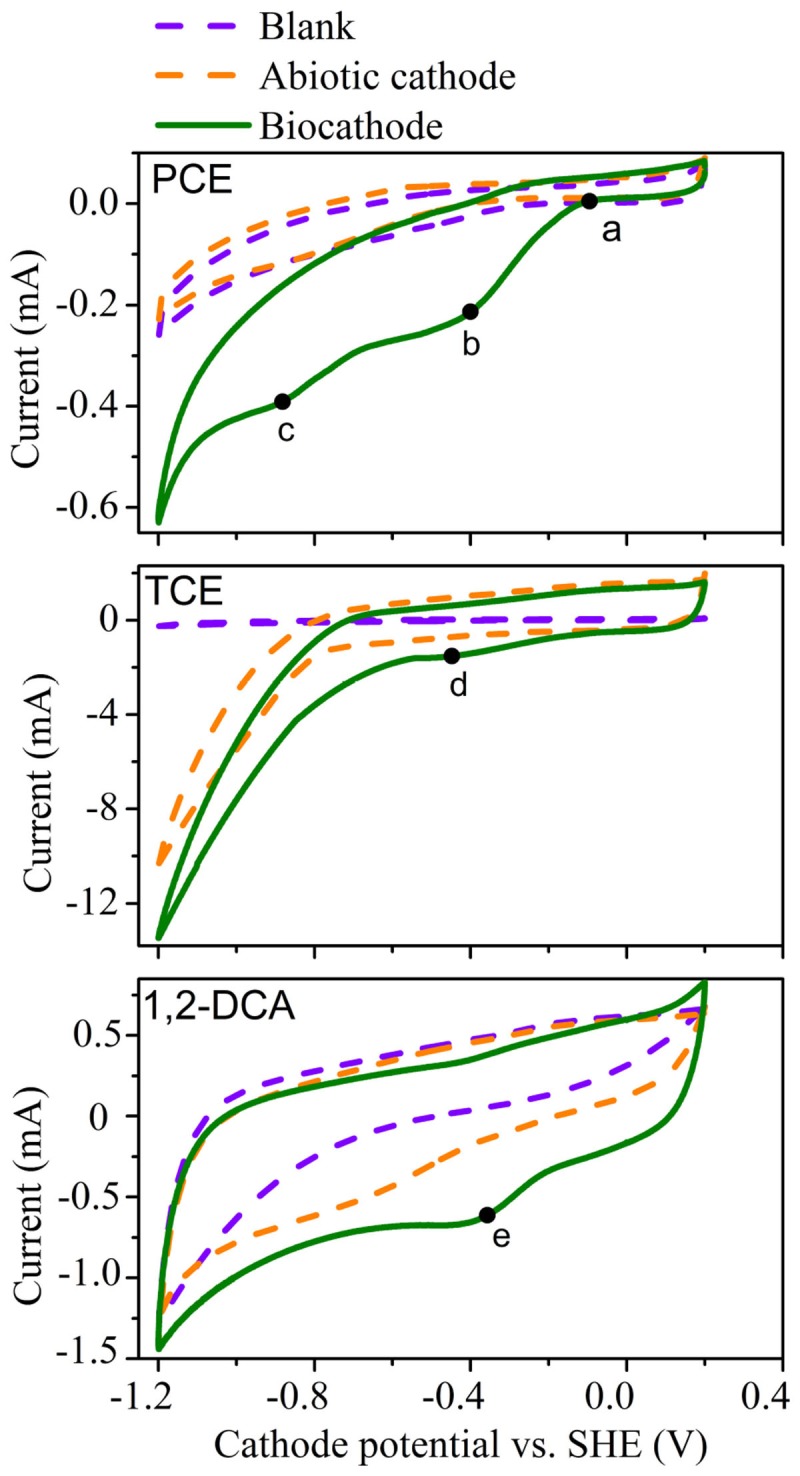
Cyclic voltammogram curves for the dechlorination of PCE, TCE, and 1,2- DCA in acclimated systems with biocathode, abiotic cathode, and blank control.

CV tests were employed to investigate the feasibility of the cathodic dechlorination of PCE, TCE and 1,2-DCA, respectively (**Figure 2**). The reduction of PCE on the biocathode started at around −0.10 V (a) and the peak potentials for PCE were observed at about −0.40 V (peak a) and −0.82 V (peak b), respectively. These results suggested that there were two main cathodic reactions for PCE degradation. Compared to the CV profile of blank control and abiotic cathode for PCE, the CV of biocathode revealed the much higher positive onset potential (−0.10 V vs. −0.34 V) with the increase in cathodic current for PCE degradation. Based on the further negative scanning on cathode potential, about five times higher cathodic current area was observed in biocathode than that in the abiotic cathode, indicating the improved electrochemical activity for PCE reduction below −0.2 V (**Figure 2**). The enhancement of cathodic current and the positive shift of onset potential for the cathodic current indicated that the cathodic biofilm catalyzed the bioelectrochemical PCE reduction.

Similar enhancement of cathodic current was observed during the reduction of TCE, and no increase in cathodic current was observed before −1.2 V without CAHs (blank control), which strongly suggested the catalytic effects of the cathodic biofilm for the bioelectrochemical reduction of TCE. Meanwhile, for 1,2-DCA reduction, current area of biocathode is about 1.5 times of that of the abiotic cathode, demonstrating the improved reduction activity by the biocatalyzed cathode. The redox peaks (b, d, and e) for the three CAHs are observed at the similar cathode potential interval from −0.2 to −0.4 V, highly related to the effective dechlorination performance at a similar cathode potential of −0.26 V. The CV results together indicated that cathodophilic microbes enhanced the biocathodic electron transfer for the catalysis of CAHs reduction.

### CAHs Dechlorination Metabolites and Pathways Under the Different Modes

**FIGURE 3:**
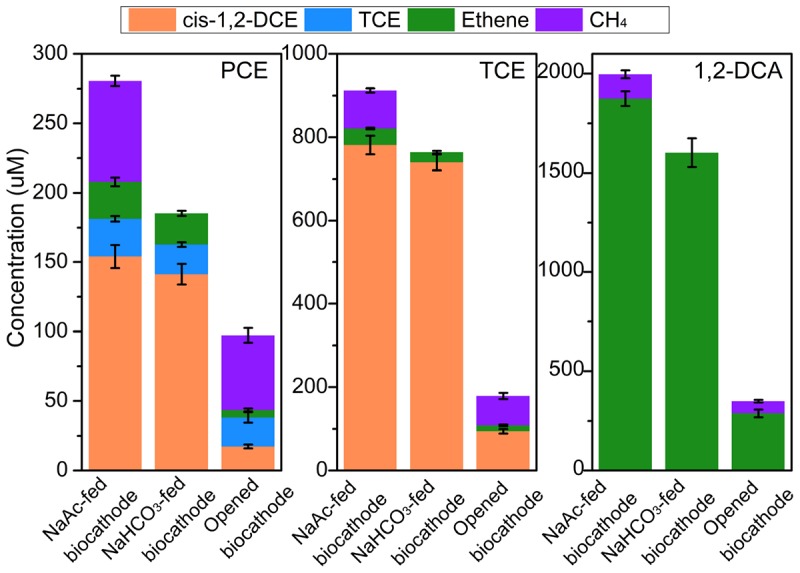
The concentration of major dechlorination metabolites and methane during PCE, TCE, and 1, 2-DCA dechlorination under different operational modes in the acclimated biocathode system at 24 h. Negligible metabolites (<10 μM) including *trans*-DCE, 1,1-DCE, and VC were not included.

There were no obvious dechlorination products of all three CAHs detected at the abiotic cathode of −0.26 V. The dechlorination of PCE in NaAc-fed biocathode, NaHCO_3_-fed biocathode and opened circuit biocathode resulted in the generation of lesser-chlorinated compounds including mainly TCE, *cis*-1,2-DCE and ethene at 24 h (**Figure 3**). The accumulation of TCE with the concentration of 20.8–27.3 μM occurred at all biocathodes. However, TCE accounted for 30.7 ± 5.4% of the total CAH dechlorination products in opened circuit biocathode, which was about three times of that in NaAc-fed (11.0 ± 0.8%) and NaHCO_3_-fed biocathodes (10.1% ± 0.7%), respectively. The lower proportion of TCE in biocathode demonstrated the electrochemical stimulation could accelerate the PCE dechlorination. In addition, *cis*-1,2-DCE was identified as the major dechlorination metabolites in both the NaAc-fed and NaHCO_3_-fed biocathodes, with recovery rates of 62.1 ± 3.4 and 66.0 ± 4.7%, respectively. However, in contrast to the larger accumulation of *cis*-DCE, the recovery rate of ethene was 2.3–4.8 times lower. Besides, negligible 1,1-DCE, *trans*-1,2-DCE, or VC was detected throughout PCE dechlorination. Similarly, TCE was prominently dechlorinated to *cis*-1,2-DCE (R_cis–DCE_, 50.4–79.8%) and weakly to ethene (R_ETH_, 2.6–7.6%). In comparison, the removed 1,2-DCA was almost dechlorinated to ethene via dichloroelimination. For the three CAHs, the similar dechlorination pathways were observed under biocathode and the sole microbial process, indicating electrochemical stimulation only accelerated the dechlorination rates and did not change the pathway in biocathode systems.

Phylogenetically diverse bacteria can dechlorinate PCE and TCE to *cis*-1,2-DCE (i.e., *D. mccartyi* and *D. restrictus* strains; *Geobacter* spp., *Desulfitobacterium* spp., and *Sulfurospirillum* spp.) (Holliger et al., 1998; Suyama et al., 2001; Luijten et al., 2003; Strycharz et al., 2008; Fletcher et al., 2011; Lee et al., 2013; Löffler et al., 2013). However, so far, only *Dehalococcoides* species includes bacteria capable of further dechlorination past *cis*-1,2-DCE to ethene (i.e., *D. mccartyi* strain 195 and 11a) (Fletcher et al., 2011; Lee et al., 2013; Löffler et al., 2013). The accumulated *cis*-1,2-DCE could be further oxidized using anode or trace oxygen as electron acceptor after sequential dechlorination by the cathode in the configurations such as flow-through bioelectrochemical barrier and bioelectric well (Lohner et al., 2011; Verdini et al., 2015; Wang et al., 2015a; Palma et al., 2018). The observed dechloroelimination of 1,2-DCA was very similar to that in previous research on bioelectrochemically assisted reductive dechlorination of 1,2-DCA by a *Dehalococcoides*-enriched microbial culture (Leitão et al., 2015). The *D. ethenogenes* strain *195* and *D. mccartyi* strain BAV1 have demonstrated the ability to convert 1,2-DCA to ethene via dechloroelimination (Maymó-Gatell et al., 1999; He et al., 2003). *Dehalobacter* sp. is proved to be capable of dechlorinating 1,2-DCA to ethene in coculture with an *Acetobacterium* sp. (Grostern and Edwards, 2009). Other isolates, such as *Desulfitobacterium dichloroeliminans* strain DCA1, are capable of selectively dechlorinating 1,2-DCA but not chlorinated ethenes (De Wildeman et al., 2003; Marzorati et al., 2007).

Methane production varied obviously in different operational modes (**Figure 3**). In autotrophic biocathode, no methane was detected in the effluent, indicating the electrons released at the cathode could not be used for producing methane. This might be attributed to the fact that the cathode potential (−0.26 V) was not reducing enough to sustain the abiotic reduction of H^+^ to H_2_ (standard reduction potential of −0.41 V at pH 7) for methane production by hydrogenotrophic methanogens, or not reducing enough for electrochemical reduction of CO_2_ by electromethanogenesis (Cheng et al., 2009; Siegert et al., 2015). However, the methane production could reach 72.5–121.3 and 53.8–70.0 μM in heterotrophic biocathode and opened circuit biocathode, respectively. In heterotrophic cultivation, acetotrophic methanogens were capable of producing methane with acetate as substrate (Alvarado et al., 2014). In addition, the methane concentration in the heterotrophic biocathode was 30.3–97.9% higher than that in opened circuit biocathode, indicating electrochemical stimulation not only enhanced CAHs dechlorination activity but also enhanced methane production. There is the possibility of direct electron transfer to acetotrophic methanogens in heterotrophic biocathode (Cheng et al., 2009).

### Electron Fluxes Through Electron Balance Calculation

**FIGURE 4:**
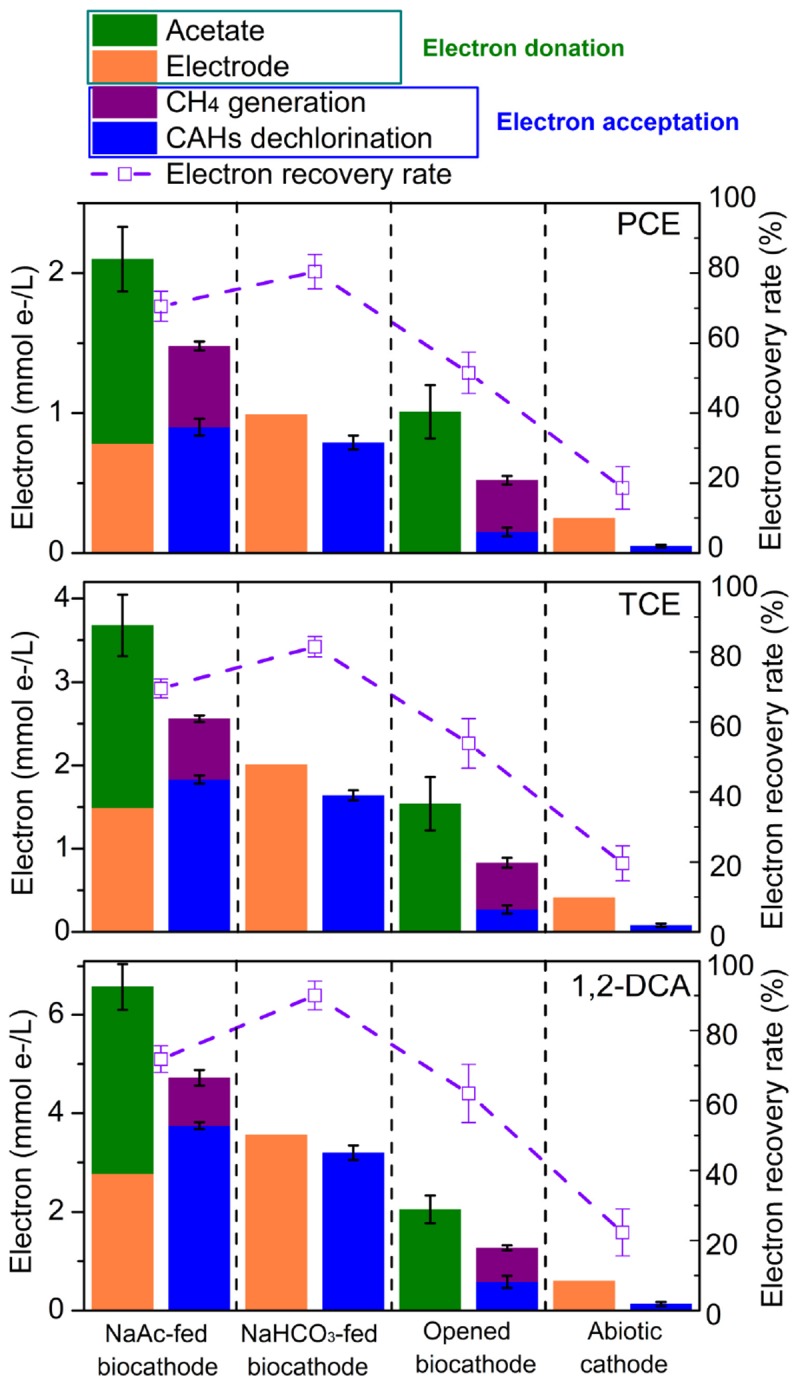
Calculated electron balances during CAHs dechlorination at 24 h under the different operational modes with cathode potential of –0.26 V.

Electron balances were conducted to better understand the accelerated dechlorination related electron transfer mechanism observed between organic substrate (acetate), electrode, and CAHs for dechlorinators and methanogens as shown in **Figure 4**. Methanogenesis was observed appeared in acetate-fed biocathodes and opened circuit biocathodes but not in NaHCO_3_-fed biocathodes or abiotic cathodes. This indicated that methanogenesis was likely supported by acetate utilized methanogens rather than by the electrode function of carbonate reduction or by the combined activities of acetogenic bacteria and acetate-utilizing methanogens (Jiang et al., 2013). Previous reports also confirmed that at roughly −0.26 V of cathodic potential, contributions of the electrode to methane generation are likely valued at less than 1% relative to the dechlorination process (Aulenta et al., 2011).

Taking dechlorinating PCE as an example, the donated electrons originated from either acetate (1.32 mmol/L) or cathodic electrode (0.78 mmol/L), respectively; among of them, 0.58 and 0.9 mmol/L of electrons should be utilized for methane generation and PCE dechlorination, respectively (**Figure 4**). As all methanogenesis originated from acetate, 43.9% of acetate should be utilized for methane generation, and 56.1% should probably be utilized for carbon source or PCE dechlorination. Similarly, 33.3% and 25.5% of acetate were, respectively, utilized for methanogenesis during TCE and 1,2-DCA dechlorination. εPCE, εTCE, and εDCA values reached 115.3 ± 8.1, 122.8 ± 5.0, and 135.4 ± 3.4%, and coulombic efficiencies of higher than 100% might be attributed to the higher contribution of acetate as an electron donor for CAHs dechlorination. In NaHCO_3_-fed biocathode, εPCE, εTCE, and εDCA approached 80.4 ± 4.9, 81.5 ± 2.9, and 90.0 ± 4.1% for PCE, TCE, and 1,2-DCA, respectively, showing that electrodes served as an alternative electron donor to efficiently promote CAHs dechlorination when both acetate and electrodes were present.

In the opened circuit biocathode, acetate worked as the sole electron donor for both CAHs dechlorination and methane generation (He et al., 2002). Under this condition, methane generation became the predominant metabolic activity competing with CAHs dechlorination. For example, in PCE dechlorination system, 14.9 and 36.6% of acetate were, respectively, utilized for dechlorination and methane generation (**Figure 4**), and the other parts (48.5%) might be attributed to its use for cell growth, adsorption onto the cathodic biofilm and measurement error. A comparison of electronic usage patterns observed from the closed and opened circuit biocathodes also showed CAHs dechlorination and methane generation were significantly enhanced by electrical stimulation. εPCE (18.6 ± 6.1%), εTCE (19.6 ± 5.0%), and εDCA (22.3 ± 6.7%) of the abiotic cathode were much lower than those of biocathodes (**Figure 4**), indicated that CAHs dechlorination through single electrocatalysis was far less efficient, correlating with the poor performance of the abiotic cathode (**Figure 1**). Thus, in the electrochemically stimulated dechlorination systems, electrodes likely served as persistent external electron donors that might be responsible for CAHs dechlorination, while acetate in cathodes acted as an internal electron donor and carbon sources largely contributing to methanogenesis and cell growth and contributing little to CAHs dechlorination. It should be noticed the planktonic biomass in catholyte besides the cathodic biofilm may still undergo dechlorination, methanogenesis, and electrochemical activity (Leitão et al., 2015). Contribution from planktonic biomass (not attached to the electrodes) on the enhanced dechlorination activity could be further verified through dechlorination activity or bacterial community structure and function analysis.

### Metabolic Processes in Biocathode Performing CAHs Dechlorination

**FIGURE 5:**
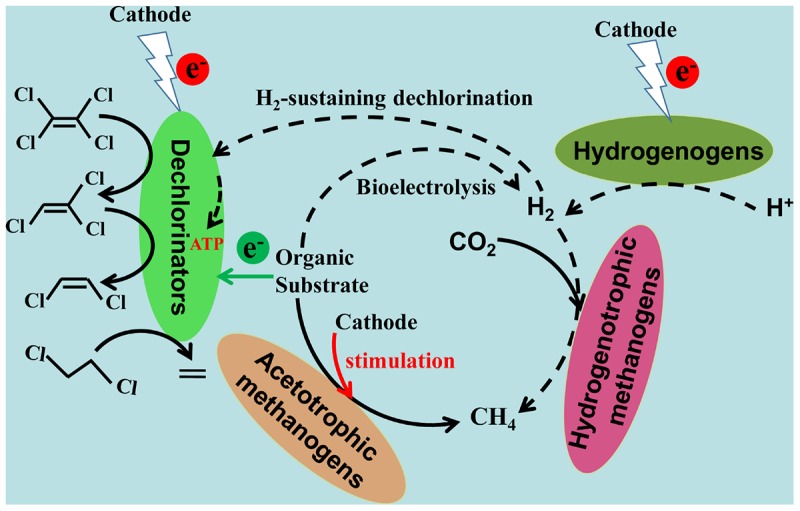
A concept model for the enhanced CAHs dechlorination in biocathode bioelectrochemical systems. Black solid and dotted arrows indicate the process verified by this study and the presumed process based the previously reported studies, respectively.

Based on the dechlorination metabolites, pathways, and electron balance, a concept model for the enhanced CAHs dechlorination in biocathode BESs was proposed as shown in **Figure 5**. Metabolic processes involving electron transfer during CAHs dechlorination in biocathode mainly included dechlorination, hydrogen, and methane production. The electron transfer from the electrode to dechlorinators via the intermediate electrolytic generation of molecular hydrogen has been proved as an important pathway, which is largely dependent on the applied cathodic potential (Aulenta et al., 2011; Lai et al., 2017). In this study, with the cathode potential of −0.26 V, the utilization of electrons from electrode rather than generated hydrogen would be the primary dechlorination metabolic pathway. The pathway of electrode serving as the electron donor for dechlorinators also has been demonstrated in previous studies (Strycharz et al., 2008, 2010).

As far, H_2_-sustaining CAH dechlorination was considered with a much lower electron utilization efficiency than the direct capture of electrons from electrode by cathode-utilizing dechlorinators, attributed to the low H_2_ solubility and H_2_ hydrolysis capacities (Luijten et al., 2004; Leitão et al., 2015). Besides, the alkali conditions surrounding electrode caused by electrolytic hydrogenation or H_2_-driven side bio-reactions like methanogenesis would be other accounts for the reduced dechlorination activities (Aulenta et al., 2011; Verdini et al., 2015).

In this study, the pathway with acetate as a direct electron donor for CAHs dechlorination existed in heterotrophic biocathode. In addition, an electron transfer pathway from acetate to hydrogen could be introduced by bioelectrolysis (Guo et al., 2017), competing with dechlorination for electrons provided by acetate. Methane production by acetotrophic and hydrogenotrophic methanogens was considered as an electron sink in heterotrophic biocathode, ultimately reducing the fraction of electrons provided from acetate and electrode that were used for CAHs dechlorination.

According to the electron balance analysis for biocathode (**Figure 4**), it could be speculated that electron fluxes for bacterial growth (ATP generation) were another electron sink (Virdis et al., 2009; Kracke et al., 2015; Ueki et al., 2018). The electron fluxes for CAH dechlorination could originate from cathode, organic substrate and hydrogen (more negative potential than −0.41 V for abiotic reduction of H^+^ to H_2_). The various pathways of electron supply in biocathode BES might be responsible for the enhanced CAHs dechlorination. The activities of dechlorinators, methanogens and hydrogenogens could be promoted by electrochemical stimulation.

## Implications

The study is the first to reveal the electron fluxes in biocathode BES performing dechlorination of PCE, TCE, and 1,2-DCA with a constant cathode potential of −0.26 V. The biocathode had significantly higher dechlorination efficiency than the opened circuit and abiotic cathode, respectively, indicating the improved CAHs dechlorination capacities. Cyclic voltammetry analysis further revealed the superior dechlorination potential of formed the cathodic biofilm by the enhanced cathodic current and the positive shift of onset potential. The dechlorination pathway in biocathode systems was consistent with the pure microbial system, that with *cis*-1,2-dichloroethene and ethene as the primary products for PCE/TCE and 1,2-DCA, respectively. Besides, methane was the main by-product of heterotrophic biocathode and methane production was enhanced to some extent by electrochemical stimulation. The various electron fluxes originating from cathode and acetate might be responsible for the enhanced CAHs dechlorination, and while, methane generation and bacterial growth would probably reduce the fraction of electrons provided for CAH dechlorination. The study gave suggestions on electron distribution and transfer in systems for the enhanced dechlorination of CAHs by electrochemical stimulation.

## Author Contributions

ZL, AW, and FC designed the study. FC and ZL wrote this manuscript. JY and FC did most of the test. BL, CH, and WC helped data analysis. AW and JN helped to improve the quality of this manuscript.

## Conflict of Interest Statement

The authors declare that the research was conducted in the absence of any commercial or financial relationships that could be construed as a potential conflict of interest.
